# Problematic Use of Mobile Phones in Australia…Is It Getting Worse?

**DOI:** 10.3389/fpsyt.2019.00105

**Published:** 2019-03-12

**Authors:** Oscar Oviedo-Trespalacios, Sonali Nandavar, James David Albert Newton, Daniel Demant, James G. Phillips

**Affiliations:** ^1^Centre for Accident Research and Road Safety-Queensland (CARRS-Q), Queensland University of Technology (QUT), Brisbane, QLD, Australia; ^2^Institute of Health and Biomedical Innovation (IHBI), Queensland University of Technology (QUT), Brisbane, QLD, Australia; ^3^Department of Industrial Engineering, Universidad del Norte, Barranquilla, Colombia; ^4^School of Psychology, The University of Queensland, Brisbane, QLD, Australia; ^5^Faculty of Health, Australian Centre for Public and Population Health Research, University of Technology Sydney, Ultimo, NSW, Australia; ^6^School of Public Health and Social Work, Faculty of Health, Queensland University of Technology (QUT), Brisbane, QLD, Australia; ^7^Psychology Department, Auckland University of Technology, Auckland, New Zealand

**Keywords:** internet addiction, cell phone, human engineering, human-computer interaction, driver behavior, road safety

## Abstract

Rapid technological innovations over the past few years have led to dramatic changes in today's mobile phone technology. While such changes can improve the quality of life of its users, problematic mobile phone use can result in its users experiencing a range of negative outcomes such as anxiety or, in some cases, engagement in unsafe behaviors with serious health and safety implications such as mobile phone distracted driving. The aims of the present study are two-fold. First, this study investigated the current problem mobile phone use in Australia and its potential implications for road safety. Second, based on the changing nature and pervasiveness of mobile phones in Australian society, this study compared data from 2005 with data collected in 2018 to identify trends in problem mobile phone use in Australia. As predicted, the results demonstrated that problem mobile phone use in Australia increased from the first data collected in 2005. In addition, meaningful differences were found between gender and age groups in this study, with females and users in the 18–25 year-old age group showing higher mean Mobile Phone Problem Use Scale (MPPUS) scores. Additionally, problematic mobile phone use was linked with mobile phone use while driving. Specifically, participants who reported high levels of problem mobile phone use, also reported handheld and hands-free mobile phone use while driving.

## Introduction

The use of smartphones has continued to amplify over the years, with the total number of smartphone users worldwide projected to surpass the 2.5 billion mark in 2019 (1). In Australia, approximately 88% of the population owns a smartphone, thus making Australia one of the foremost adopters of such technology (2). Other Western countries such as the United States of America, for instance, have found approximately 64% of its population to use a smartphone as of 2017 (3). Additionally, in developing countries such as India, smartphone ownership rates were expected to reach 36% by 2018 (4), and 46.1% in Sub-Saharan Africa by the end of the same year (5). Such high ownership rates in Australia and across the globe can be attributed to scale manufacturing practices, socio-economic factors, and advancements in technology over the years.

Mobile phone devices are now commonly referred to as “smartphones,” as they offer greater functionality and numerous gratifications over and above traditional phones (6). Initially designed for basic functionalities such as calling and texting, smartphones have significantly changed to now include engagement in a wide range of activities such as navigating social media (e.g., Instagram®), browsing the internet, and playing games, all in one device (7, 8). As social media and other applications are primarily accessed via smartphones, communication is often viewed as the most prevalent use of smartphones today (8, 9). Thus, one would expect such a powerful piece of technology to have struck a chord within the community and have a tremendous social impact on modern society, particularly among populations with the highest use such as young people (8). For the purposes of this paper, and from here on out, the term “mobile phone” was used when referring to “smartphone.”

### Benefits of Mobile Phone Technology

Mobile phones serve plenty of beneficial uses, some of which have the potential to increase an individual's quality of life. According to Burckhardt and Anderson (10), quality of life is determined by five dimensions: material and physical well-being, relationships with other people, social, community and civic activities, personal development and fulfillment, and recreation. Based on these dimensions, one can assert that mobile phones promote physical well-being through helping users access help in the case of an emergency, and encourages users to form and maintain relationships with others (e.g., family or partners) via social media networks (11). Mobile phones can also promote engagement in social, community and civic activities, enabling users to support social causes such as reducing fuel consumption while driving (12), in addition to promoting personal development and fulfillment through offering users a plethora of information on a variety of topics (via websites, apps). Finally, mobile phones can also help users engage in recreational activities through games and music. While such research highlights the positive contribution made by mobile phones to modern society, problematic or excessive mobile phone use can alternatively contribute to an array of adverse outcomes.

### Negative Consequences of Mobile Phone Use

Negative and adverse outcomes associated with problematic mobile phone use have caught the attention of researchers around the world. Most of the research in this area continues to assume that some individuals are having maladaptive relationships with their mobile phone devices. This phenomenon is evident in cases wherein certain individuals were reported to have endured feelings of stress or separation anxiety when they were unable to use their phone (13, 14). In addition, some individuals have also expressed difficulties in disconnecting from smartphone use particularly due to its utility and usefulness in filling gaps during the performance of mundane tasks (15). According to (11), problematic or excessive mobile phone use refers to an individual's inability to control their usage of their mobile phone which, in turn, leads to adverse consequences in their everyday life. On a personal level, such consequences may relate to financial problems, sleep disturbances, attentional and learning impairments in educational settings, excessive sedentary behavior, and the deterioration of personal relationships (11, 16–20). It should also be noted, however, that although certain parallels do exist between addiction/dependency syndromes and excessive mobile phone use, this paper will refrain from referring to this behavior as “addictive,” as the core symptoms are vastly different from classically recognized and defined addictions such as substance-use disorders or gambling (6, 21). Nonetheless, it is apparent that in some instances excessive use of mobile phones can be problematic, with greater or more frequent use creating higher functional impairments (21).

When an individual uses a mobile phone in physically hazardous situations, such use can be considered extremely or highly problematic. The use of a mobile phone while driving, for instance, is an example of problem mobile phone use as it falls toward the end of the mobile phone use spectrum or, in other words, creates more serious health and safety implications. Greater problematic mobile phone use is apparent in Australia, with 61% of active Australian drivers having reported using their mobile phone while driving despite the unlawfulness and dangers surrounding this behavior (22). Another example is using a mobile phone while crossing the road. Research shows that at least a quarter of pedestrians cross the road while engaged in visual-manual interactions such as texting and browsing on a mobile phone (23). Given the increasing risks of phone use while walking, some jurisdictions (Honolulu; Mont Clair; Stamford) now prohibit the use of mobile phones while using cross-walks (24).

Additionally, due to the advanced functionality of mobile phones, an increase in usage may also result in individuals being subjected to security issues such as location tracking, access to personally identifiable information (through software apps), and mobile malware, all of which can increase users risks of falling victim to identity theft or robbery (25). Similarly, as the capabilities of mobile phones continue to increase, certain individuals may be more prone to abusing functions offered by these devices (e.g., Freemium games) and developing addictive behaviors such as pathological gambling (26).

### Problem Mobile Phone Use: Scales and Questionnaires

The need to study these potentially negative impacts of mobile phone misuse has required the development of a number of tools to identify maladaptive mobile phone use. Specifically, a range of psychometrically sound scales/questionnaires has been developed to measure problematic mobile phone use. The Problematic Mobile Phone Use Questionnaire [PMPUQ; (27)] was developed to measure problematic phone use based on four factors: prohibited use, dangerous use, dependence symptoms, and financial problems associated with its use. Additionally, the Problematic Cellular Phone Use Questionnaire [PCPU-Q; (28)] is another scale that has been developed to measure problematic cell phone use based on the taxonomies of substances use dependence (e.g., symptoms, functional impairment). However, one of the pioneering tools used in this area of research, the Mobile Phone Problem Use Scale [MPPUS; (29)], has ubiquitously been used in the literature for many years. According to (29) problem mobile phone use in the MPPUS is measured based on potential predictors of behavioral and technological addiction. The MPPUS considers issues involving tolerance, withdrawal, craving, negative life consequences (e.g., social, financial), and escaping from other problems. This scale has demonstrated high levels of validity and reliability [α = 0.93; 26; α = 0.94; (30)], has been used in several studies across different countries, and is considered to be a highly useful tool in measuring a universal score of problematic mobile phone use (11).

### The Present Study

Since the development of the MPPUS in 2005, countries around the world have witnessed the speed at which mobile phone technology has changed over the past decade. Hence, it is of utmost importance to revisit and update the literature, in order to determine whether problematic mobile phone use has become a pervasive issue in today's world. Currently, there is a gap in the literature regarding the current trends of problem mobile phone use in Australia. In addition, it is also unclear whether problem mobile phone use, as originally defined by Bianchi and Phillips (29), has changed since 2005. Thus, in order to address these gaps in the literature, the present study has two main objectives:

- The first objective is to investigate the current problem mobile phone use in Australia and its potential implications for road safety.
- The second objective is to use the original 26 study to identify trends of change related to mobile phone misuse in the Australian population. This time comparison is relevant given the changing nature of mobile phones and growing pervasiveness of mobile phones in Australian society.

In comparisons of samples collected in 2005 and 2018, it is expected that the proportions of the sample reporting high levels of problem mobile phone use has increased from the first data collected in 2005. The following sections describe the methods used in this study.

## Methods

### Participants

The present study included a total of 709 participants (365 males and 344 females). Participants' ages ranged between 18 and 83 years, with the highest number of participants (*n* = 135; 19%) belonging to the 56–65 year-old age group (see Table 1). Based on eligibility criteria, all participants were aged 18 years and over, resided in Australia and owned/used a mobile phone. Participants also reported their level of education, with the majority having completed or were currently enrolled in university (*n* = 473; 66.7%), followed those by who completed or were currently enrolled in TAFE (*n* = 114; 16.1%), finished year 12 high schooling (*n* = 79; 11.1%), and completed junior year 10 high schooling or equivalent (*n* = 43; 6.1%).

**Table 1:** Personal Characteristics of Participants in the 2005^a^ and 2018 studies conducted in Australia.

		**2005^a^**	**2018**
Sex	Male	62 (31.8%)	365 (51.5%)
	Female	132 (67.7%)	344 (48.5%)
	Missing	1 (0.5%)	–
Age Groups	18–25 years	38 (19.5%)	109 (15.4%)
	26–35 years	71 (36.4%)	116 (16.4%)
	36–45 years	43 (22.1%)	98 (13.8%)
	46–55 years	30 (15.4%)	114 (16.1%)
	56–65 years	3 (1.5%)	135 (19.0%)
	66–75 years	7 (3.6%)	123 (17.3%)
	76–85 years	1 (0.5%)	14 (2.0%)
	Missing	2 (1%)	–
	Total	195	709

a *Bianchi and Phillips (29)*.

With the aim to generate a wide and large sample, potential participants were recruited throughout Australia using various methods, including a Queensland University of Technology (QUT) media release, social media ads on Facebook® and Twitter®, and emails from local insurance companies. Participants provided consent through the online submission of the survey.

### Measures

The present study utilized the 27-item Mobile Phone Problem Use Scale [MPPUS; (29)] as the main tool of analysis. Items in the MPPUS (29) assess symptoms of behavioral and technological addiction such as issues of tolerance, escaping from problems, withdrawal, craving, and negative life consequences (e.g., financial, work, social, and family). In addition, the MPPUS (29) also consisted of items that examined loss of control over mobile phone usage (e.g., “I find it difficult to switch off my mobile phone”), time spent on mobile phone-related activities, and social motivational aspects of mobile phone use (e.g., “All my friends own a mobile phone”). Participants responded to questions on a 10-point Likert-type scale, ranging from “not at all” (1) to “extremely true” (10).

The MPPUS (29) was validated based on moderately strong positive correlations reported between the scale and other measures of mobile phone use such as self-reported time spent using the mobile phone during the week, average monthly expenditure, and the number of calls made to people on a regular basis. In addition, good construct validity for the scale was also determined based on moderate correlations found between the MPPUS (29) and the MMPI-2 Addiction Potential Scale [APS; (31)], an established scale known for measuring addiction.

In addition to the MPPUS, participants were asked about their handheld and hands-free mobile phone use while driving a moving vehicle based on scales designed for this study. The questions pertained to the frequency with which they performed visual-manual interactions (e.g., “You performed a visual manual task on your mobile phone”; α = 0.81) and hands-free conversations (e.g., “You had a phone conversation using an in-car audio system or Bluetooth”; α = 0.76) while driving. Participants responded to questions on a 7-point Likert-type scale, ranging from “never” (1) to “always” (7).

### Data Analysis

#### Problem Mobile Phone Use in the 2018 Sample

The data analysis plan was designed to meet two objectives, the first objective being to investigate the current problem mobile phone use in Australia and its potential implications for road safety. To meet this objective, the following steps were taken.

Firstly, the reliability and validity of the MPPUS (29) were analyzed to understand the current psychometric properties. As explained earlier, the MPPUS was designed and validated in Australia by Bianchi and Phillips (29). However, societal and technological changes surrounding mobile phone use in the last 10 years necessitates confirmation of the psychometric properties, factorial structure and internal consistency of the MPPUS (29). To assess the factorial structure, a statistical technique known as an exploratory factorial analysis was conducted (via principal components analysis) to replicate the analysis in the original study. According to Grant & Fabrigar (32), this analysis is often used to reduce data by analyzing the relationship between many variables through a small number of factors. Finally, the reliability of the MPPUS was evaluated using measures of internal consistency by means of Cronbach's alpha coefficient.

Secondly, the psychometric properties of the MPPUS were confirmed: the prevalence, user categories, and cut-off points in the MPPUS were determined following the methodology developed by de-Sola et al. (30). Specifically, four categories were created: 5% of mobile phone users are problem phone users, 15% are at risk of being problem users, 65% are regular phone users, and 15% are occasional phone users. Differences by sex, age, education level, and mobile phone distracted driving among phone user categories were studied using ANOVA and correlation analyses.

Thirdly, the influence of socio-demographic and mobile phone distracted driving factors on the user categories was studied using logistic regression, a statistical analysis which involves determining the probability of an outcome through its relationship to one or more predictors (33). The statistical model predicted associations with two user categories: normal phone users (the sum of casual and habitual and regular users) and users with problematic phone usage (including the sum of at risk users and problem users).

#### Problem Mobile Phone Use Comparisons Between 2005 and 2018 Samples

The second objective was to use the original Bianchi and Phillips (29) study to identify trends of change related to mobile phone misuse across the Australian population. To meet this objective, differences between the 2005 and 2018 MPPUS samples in Australia were calculated. An item-level analysis was conducted using the Mann-Whitney *U* test. This nonparametric test is used to compare two independent samples (34). In this study, the Mann-Whitney U test was used to establish differences between the 2005 and 2018 samples by age and gender. Four age groups were defined: 18–25 years, 26–35 years, 36–45 years, and 46+ years. These groups were chosen due to the small number of participants aged 46+ in the 2005 study. Additionally, to illustrate changes in the prevalence of larger responses over time, the percentage of participants who marked a value of 6 or higher in each item was calculated for each age and gender and compared. Statistical analyses were conducted through the IBM SPSS Statistics software (version 23).

## Results

### Problem Mobile Phone Use in the 2018 Sample

#### A Retest of the MPPUS Validity and Reliability in Australia

First, a principal component analysis was conducted to validate the factorial structure of the MPPUS. During the analysis, Item 4 “All my friends own a mobile phone” was removed as it had a factor loading <0.4, while the other 26 items had factor loadings above 0.4. More recent applications of the MPPUS have also confirmed the poor performance of item 4 (35). Although the final solution showed three factors (explained variance of 62.25%), it was confirmed that there is a single factor “Problem mobile phone use” that compiles the 26 items of the MPPUS (explained variance of 49.88%). The unidimensionality of the MPPUS has been supported in the previous Australian study (29) and its replications in Britain (36) and Spain (35). The Kaiser–Mayer–Olkin index (KMO = 0.96) confirmed the adequacy of the sample, while Bartlett's test of sphericity ($\chi_{\left(325\right)}^{2}$ = 13,028.39; *p* < 0.001) supported the use of the factor analytic procedure. Five items (Item 15, Item 17, Item 25, Item 26, and Item 27) had cross-loading on one of two factors. However, cross-loading items were retained as they fitted theoretically into a single main factor where they loaded the strongest (37). Table A1 further illustrates the individual items' loadings. Second, the reliability analysis revealed an overall Cronbach's α of 0.954 for all 26 items. This value indicates that the MPPUS demonstrated high reliability in the current Australian sample.

#### Prevalence, User Categories, and Cut-Off Points in the MPPUS

Table 2 presents the mean values for each MPPUS (29) item used in Australia and other countries. A detailed analysis per item shows that Item 4 (“All my friends own a mobile phone”; *M* = 8.86; *SD* = 2.24) and Item 17 (“If I don't have a mobile phone, my friends would find it hard to get in touch with me”; *M* = 5.19; *SD* = 3.33) have the higher scores. While item 15 (“I have frequent dreams about the mobile phone; *M* = 1.37; *SD* = 1.20) had the lowest score in Australia. To put the items into context, previous studies using the MPPUS were identified in a literature review search. As can be seen in Table 2, Britain (36) consistently demonstrated higher means in comparison to Spain, Switzerland, and Australia (2018). It should also be noted that although (39) used fewer MPPUS items in the USA version, each one of their items revealed the highest means in comparison to the other countries.

**Table 2:** Mean Scores Per Item of the MPPUS (26) by Country and Year.

***Code***	***Item (Legend)***	**Spain^a^(2017)** **(16–65years)**		**Britain^b^(2014)** **(11–18years)**		**Switzerland^c^ (2015)** **(12–17years)**		**USA (2017)^d^** **(16–28years)**		**Australia (2005)^e^** **(18–85years)**		**Australia (2018)** **(18–85years)**	
		***M***	***SD***	***M***	***SD***	***M***	***SD***	***M***	***SD***	***M***	***SD***	***M***	***SD***
Item 1	I can never spend enough time on my mobile phone.	2.72	2.03	4.01	3.16	3.46	2.53	-	-	1.62	1.18	2.06	1.85
Item 2	I have used my mobile phone to make myself feel better when I was feeling down.	3.16	2.48	4.01	3.19	3.84	2.88	4.88	1.67	2.67	2.50	2.97	2.55
Item 3	I find myself occupied on my mobile phone when I should be doing other things, and it causes problems.	3.05	2.31	3.63	2.97	3.71	2.68	-	-	1.81	1.59	2.95	2.45
Item 4	All my friends own a mobile phone.	-	-	-	-	8.68	2.20	-	-	7.78	2.64	8.86	2.24
Item 5	I have tried to hide from others how much time I spend on my mobile phone.	1.87	1.74	2.82	2.76	1.95	1.97	-	-	1.35	1.09	2.26	2.19
Item 6	I lose sleep due to the time I spend on my mobile phone.	2.86	2.50	3.06	3.04	2.47	2.23	-	-	1.35	1.32	2.55	2.50
Item 7	I have received mobile phone bills I could not afford to pay.	1.98	1.92	3.10	2.83	1.37	1.40	-	-	1.88	2.06	1.53	1.59
Item 8	When out of range for some time, I become preoccupied with the thought of missing a call.	3.45	2.65	3.47	3.01	2.20	2.07	-	-	2.15	1.80	2.01	1.83
Item 9	Sometimes, when I am on the mobile phone and I am doing other things, I get carried away with the conversation and I don't pay attention to what I am doing.	3.47	2.45	3.83	3.08	2.69	2.21	-	-	2.80	2.39	2.49	2.07
Item 10	The time I spend on the mobile phone has increased over the last 12 months.	3.41	2.64	4.15	3.28	4.90	3.23	-	-	3.76	2.98	3.15	2.65
Item 11	I have used my mobile phone to talk to others when I was feeling isolated.	3.91	2.82	4.17	3.26	3.95	3.03	5.65	1.43	3.31	2.75	3.54	2.97
Item 12	I have attempted to spend less time on my mobile phone but am unable to.	2.29	2.04	2.96	2.76	2.38	2.16	-	-	1.84	1.77	2.42	2.24
Item 13	I find it difficult to switch off my mobile phone.	3.58	3.01	2.89	2.91	2.56	2.58	-	-	3.06	3.06	2.99	2.75
Item 14	I feel anxious if I have not checked for messages or switched on my mobile	2.96	2.38	3.51	3.05	2.74	2.47	4.43	1.80	3.10	2.51	2.88	2.60
Item 15	I have frequent dreams about the mobile phone.	1.53	1.48	2.31	2.48	1.25	1.10	-	-	1.11	0.49	1.37	1.20
Item 16	My friends and family complain about my use of the mobile phone.	2.22	2.05	2.80	2.75	2.98	2.62	-	-	1.42	1.14	1.92	1.90
Item 17	If I don't have a mobile phone, my friends would find it hard to get in touch with me	4.53	2.93	4.72	3.37	4.94	3.13	-	-	4.35	3.09	5.19	3.33
Item 18	My productivity has decreased as a direct result of the time I spend on the mobile phone.	1.97	1.83	2.58	2.52	2.09	1.65	-	-	1.42	1.03	2.43	2.32
Item 19	I have aches and pains that are associated with my mobile phone use.	1.85	1.82	2.35	2.46	1.43	1.30	-	-	1.42	1.27	1.85	1.85
Item 20	I find myself engaged on the mobile phone for longer periods of time than intended.	2.73	2.40	2.99	2.80	3.30	2.53	-	-	2.48	2.24	3.15	2.73
Item 21	There are times when I would rather use the mobile phone than deal with other more pressing issues.	2.04	1.87	3.11	2.86	4.14	2.84	-	-	1.68	1.53	2.99	2.70
Item 22	I am often late for appointments because I'm engaged on the mobile phone when I shouldn't be.	1.70	1.57	2.53	2.57	1.45	1.14	-	-	1.35	1.18	1.62	1.49
Item 23	I become irritable if I have to switch off my mobile phone for meetings, dinner engagements, or at the movies.	1.70	1.66	2.81	2.83	1.62	1.42	3.16	1.81	1.51	1.43	1.63	1.59
Item 24	I have been told that I spend too much time on my mobile phone.	2.22	2.16	2.92	2.86	2.86	2.68	-	-	1.57	1.69	2.01	2.01
Item 25	More than once I have been in trouble because my mobile phone has gone off during a meeting, lecture, or in a theater.	2.20	2.12	3.27	3.07	1.90	1.93	-	-	1.98	2.01	1.72	1.55
Item 26	My friends don't like it when my mobile phone is switched off.	2.78	2.49	3.45	3.09	3.02	2.60	-	-	3.11	2.69	2.47	2.36
Item 27	I feel lost without my mobile phone.	2.74	2.36	3.75	3.32	2.95	2.63	-	-	2.99	2.74	3.17	2.75

*M, Mean; SD, Standard Deviation*.

a *de-Sola et al. (30)*.

b *Lopez-Fernandez et al. (36)*.

c *Foerster et al. (38)*.

d *Kruger and Djerf (39)*.

e *Bianchi and Phillips (29)*.

Mobile phone users in the 2018 Australian sample were categorized based on the criteria by de-Sola et al. (30) as specified in section Data Analysis, using the 26-MPPUS score (see Table 3). A total of four user categories were identified: Casual Users, Habitual and Regular Users, At Risk Users, and Problem Users.

**Table 3:** Average Scores and Prevalence by User Categories of the 26-item MPPUS in Australia (2018).

**User categories**	***M***	***SD***	**Median**	**Maximum score**	**Minimum score**	**%**
Normal users (the sum of casual users and habitual and regular users)	48.48	20.36	41.00	98.00	26.00	80.4%
Casual users	27.58	1.64	27.00	30.00	26.00	18.2%
Habitual and regular users	54.60	19.23	49.00	98.00	31.00	62.2%
Problematic users (the sum of at risk users and problem users)	134.35	28.81	128.00	260.00	99.00	19.6%
At risk users	120.76	14.22	118.00	150.00	99.00	14.7%
Problem users	174.74	22.63	167.00	260.00	152.00	4.9%

*M, Mean; SD, Standard Deviation*.

##### Prevalence by sex

The ANOVA revealed significant differences in the mean 26-item MPPUS scores between males and females in the study [*F*_(1, 707)_ = 19.78, *p* < 0.001], with the females reporting more problematic mobile phone use (*M* = 72.23, *SD* = 41.36) as opposed to the males (*M* = 58.80, *SD* = 39.07). This finding is consistent with the distribution of sex across user categories, which revealed that 15.4% of males and 24.1% of females are Problematic Users.

##### Prevalence by age

Significant differences were found between the three age groups with respect to average problematic mobile phone use [F_(2, 706)_ = 83.19, *p* < 0.001], with the younger population of 18–24 year old participants reporting the highest 26-item MPPUS mean score (*M* = 95.90, *SD* = 45.58) and the older population of over 60 year old participants reporting the lowest mean score (*M* = 41.85, *SD* = 22.97). This finding is consistent with the distribution of age across user categories, such that the proportion of Problematic Users within the 18–24 year old age group was 40.9%, within the 25–59 year old age group was 23.5%, and 60 years and over was 3.2%. Significant negative correlations were found between the 26-item MPPUS and age (*r* = −0.506, *p* < 0.001), such that older participants report less problematic mobile phone use.

##### Prevalence by education level

The participants' level of education was associated with their problematic mobile phone use, with the ANOVA revealing a significant difference in the 26-item MPPUS scores between the four education levels [*F*_(3, 705)_ = 13.83, *p* < 0.001]. Overall, the participants who had completed or were currently enrolled in university (*n* = 473) reported the greatest mean 26-item MPPUS score (*M* = 71.81, *SD* = 42.63) and significantly differed from participants who had completed Year 10, finished High School, and who had completed or were currently enrolled in a TAFE course. Participants who had completed Year 10 had the lowest mean MPPUS score (*M* = 42.67, *SD* = 24.94).

##### Relationship between MPPUS and mobile phone use while driving

To understand the impact that problematic phone use has on health, this study explored the relationship between the 26-item MPPUS and mobile phone use while driving. On average, participants reported more hands-free mobile phone use of mobile phones whilst driving (*M* = 3.06, *SD* = 1.59), compared with handheld mobile phone interactions whilst driving, such as texting, browsing, etc. (*M* = 1.87, *SD* = 1.08). With regards to specific items examining handheld phone use, 46% of participants reported having looked continually at the phone for more than two seconds; while 42% reported that they have performed a visual-manual task on their mobile phone (e.g., texting, browsing, or emailing) and have monitored/read conversations without writing back. In relation to the items assessing hands-free conversations, 68% reported having had a conversation using a hands-free device (e.g., headset) and/or an in-car audio system or Bluetooth, with 62% reporting having phone conversations while driving without holding the phone in their hands (e.g. phone on loudspeaker).

The ANOVA found significant differences between handheld use [*F*_(3, 705)_ = 62.50, *p* < 0.001] and hands-free use [*F*_(3, 705)_ = 10.80, *p* < 0.001] while driving based on user categories. *Post-hoc* comparison tests revealed that Problem Users significantly differ from Casual or Habitual and Regular Users for both handheld and hands-free phones while driving, such that Problem Users engage in more handheld and hands-free mobile use whilst driving compared to Casual or Habitual and Regular Users. Finally, it should be noted that significant positive correlations were found between the 26-item MPPUS and both handheld (*r* = 0.503, *p* < 0.001) and hands-free (*r* = 0.158, *p* < 0.001) phone use while driving.

#### Predictive Variables of Problematic Users

A binary logistic regression was performed to identify the variables that predict Normal Users and Problematic Users. The independent variables used in this analysis were age, sex, level of education, and mobile phone use while driving (handheld and hands-free phone use while driving). The dependent variables comprised of the two categories used in the de-Sola et al. (30) article: Normal Users and Problematic Users. As can be seen in Table 4, the results revealed that three variables: age, handheld mobile phone use while driving, and education level, were capable of differentiating between Normal Users and Problematic Users. More specifically, it was found that an individual is more likely to be a deemed as a Problematic User if they use a handheld mobile phone while driving and belong to either the 18–24 or 25–59 year old age group. However, participants currently enrolled in or who completed TAFE education compared to those who are studying or graduated from university are less likely to be classified as Problematic Users.

**Table 4:** Logistic Regression Analysis predicting User Category.

**Variables**	**β**	***SE***	**Wald**	***df***	**Sig**.	**Exp(B)**	**95% C.I. for EXP(B)**	
							**Lower**	**Upper**
Handheld Use	0.746	0.101	54.806	1	< 0.001	2.108	1.730	2.568
Hands-free Use	−0.059	0.076	0.592	1	0.442	0.943	0.812	1.095
Age (60+ years)			24.214	2	< 0.001			
Age (18–24 years)	2.262	0.462	23.954	1	< 0.001	9.606	3.882	23.768
Age (25–59 years)	1.593	0.424	14.143	1	< 0.001	4.917	2.144	11.278
Gender (Male)	−0.170	0.222	0.585	1	0.444	0.844	0.545	1.305
Education Level (University)			7.712	3	0.052			
Education Level (Junior High School–Yr 10)	−0.381	0.648	0.345	1	0.557	0.683	0.192	2.434
Education Level (Senior High School–Yr 12)	−0.682	0.421	2.621	1	0.105	0.506	0.221	1.154
Education Level (TAFE)	−0.941	0.396	5.627	1	0.018	0.390	0.179	0.849

β,standardized regression coefficient; df, degrees of freedom; SE, standard error.

*Dependent variable: normal users = 0, problematic users = 1*.

### Problem Mobile Phone Use Comparisons Between 2005 and 2018 Samples

An item-level analysis was conducted comparing scores from the study conducted by Bianchi and Phillips (29) and the current study. As can be seen in Table 2, the average MPPUS individual item scores have increased from when the scale was first used within the Australian population in 2005. For instance, Australian participants in 2018 have been feeling, on average, more lost without their mobiles phones (Item 26, *M* = 3.17, *SD* = 2.75) in comparison to Australian participants in 2005 (*M* = 2.99, *SD* = 2.74), and have also reported experiencing, on average, more problems when occupied on their mobile phones in 2018 (Item 3, *M* = 2.95, *SD* = 2.45) compared to Australians in 2005 (*M* = 1.81, *SD* = 1.59). Similarly, Australians in 2018 have also experienced, on average, more loss of sleep due to time spent on their mobile phones (Item 5, *M* = 2.55, *SD* = 2.50), in comparison to Australian mobile phone users in 2005 (*M* = 1.35, *SD* = 1.32).

Mann-Whitney U tests of independent samples were conducted to study differences between the 2005 and original 27-item 2018 MPPUS based on sex and age. With regards to sex, average MPPUS item scores increased for eight items within the male population between 2005 and 2018 (see Table 5). In relation to the female population, the analysis revealed an increase in the average MPPUS item scores for more than half of the items in the scale between 2005 and 2018 (i.e., 15 items; see Table 5). The largest differences between the 2005 and 2018 MPPUS were found in Item 6 (“I lose sleep due to the time I spend on my mobile phone”; Δ = 0.93, *p* < 0.001) with an increase among the male population, and Item 21 (“There are times when I would rather use the mobile phone than deal with other more pressing issues”; Δ = 1.83, *p* < 0.001) with an increase among the female population. Additionally, males reported the most substantial decreases in Item 10 (“The time I spend on the mobile phone has increased over the last 12 months”; Δ = −1.48, *p* < 0.001) and Item 26 for females (“My friends don't like it when my mobile phone is switched off”; Δ = −0.66, *p* <0.01).

**Table 5:** Differences between 2005 and 2018 MPPUS based on Sex using Mann–Whitney *U* test.

**Items**		**Male**		**Female**	
		**Δ**	**Sig**.	**Δ**	**Sig**.
Item 1	I can never spend enough time on my mobile phone.	0.45	–	0.5	–
Item 2	I have used my mobile phone to make myself feel better when I was feeling down.	0.42	–	0.52	↑*
Item 3	I find myself occupied on my mobile phone when I should be doing other things, and it causes problems.	0.87	↑**	1.51	↑***
Item 4	All my friends own a mobile phone.	0.92	↑***	1.26	↑***
Item 5	I have tried to hide from others how much time I spend on my mobile phone.	0.62	↑**	1.18	↑***
Item 6	I lose sleep due to the time I spend on my mobile phone.	0.93	↑***	1.51	↑***
Item 7	I have received mobile phone bills I could not afford to pay.	−0.11	–	−0.47	↓*
Item 8	When out of range for some time, I become preoccupied with the thought of missing a call.	−0.39	↓*	0.08	–
Item 9	Sometimes, when I am on the mobile phone and I am doing other things, I get carried away with the conversation and I don't pay attention to what I am doing.	−0.6	–	−0.04	–
Item 10	The time I spend on the mobile phone has increased over the last 12 months.	−1.48	↓***	−0.08	–
Item 11	I have used my mobile phone to talk to others when I was feeling isolated.	0.01	–	0.76	↑*
Item 12	I have attempted to spend less time on my mobile phone but am unable to.	0.51	↑*	0.71	↑**
Item 13	I find it difficult to switch off my mobile phone.	−0.3	–	0.22	–
Item 14	I feel anxious if I have not checked for messages or switched on my mobile	−0.87	↓***	0.26	–
Item 15	I have frequent dreams about the mobile phone.	0.28	↑*	0.22	–
Item 16	My friends and family complain about my use of the mobile phone.	0.52	–	0.55	↑**
Item 17	If I don't have a mobile phone, my friends would find it hard to get in touch with me	−0.2	–	1.62	↑***
Item 18	My productivity has decreased as a direct result of the time I spend on the mobile phone.	0.9	↑*	1.15	↑***
Item 19	I have aches and pains that are associated with my mobile phone use.	0.23	–	0.63	↑***
Item 20	I find myself engaged on the mobile phone for longer periods of time than intended.	−0.04	–	1.35	↑***
Item 21	There are times when I would rather use the mobile phone than deal with other more pressing issues.	0.82	↑**	1.83	↑***
Item 22	I am often late for appointments because I'm engaged on the mobile phone when I shouldn't be.	0.13	–	0.37	↑**
Item 23	I become irritable if I have to switch off my mobile phone for meetings, dinner engagements, or at the movies.	−0.05	–	0.24	–
Item 24	I have been told that I spend too much time on my mobile phone.	0.46	–	0.52	↑**
Item 25	More than once I have been in trouble because my mobile phone has gone off during a meeting, lecture, or in a theater.	−0.81	↓**	−0.01	–
Item 26	My friends don't like it when my mobile phone is switched off.	−0.57	↓*	−0.66	↓**
Item 27	I feel lost without my mobile phone.	0.04	–	0.45	–

Δ = difference between item scores (2005 to 2018);

* p < 0.05;

** p < 0.01;

*** *p < 0.001*.

Additionally, concerning the age-related differences between the 2005 and 2018 MPPUS, the results revealed 18 MPPUS item scores to increase among the 26–35 and 36–45 year old age groups, and 12 MPPUS items scores to increase among the 18–25 and 46+ year old age groups (see Table 6). Furthermore, as can be seen in Table 6, the largest differences between the 2005 and 2018 MPPUS were found in Item 21 among the 18–25 year old participants (“There are times when I would rather use the mobile phone than deal with other more pressing issues”; Δ = 2.85, *p* < 0.001) and 26–35 year olds (Δ = 2.88, *p* < 0.001), Item 4 among the 36–45 year old participants (“All my friends own a mobile phone”; Δ = 2.41, *p* < 0.001), and Item 17 among the 46+ year old age group (“If I don't have a mobile phone, my friends would find it hard to get in touch with me”; Δ = 1.80, *p* < 0.001).

**Table 6:** Differences between 2005 and 2018 MPPUS based on Age using Mann–Whitney *U*-test.

**Item**		**18–25 years**		**26–35 years**		**36–45 years**		**46+** **years**	
		**Δ**	**Sig**.	**Δ**	**Sig**.	**Δ**	**Sig**.	**Δ**	**Sig**.
Item 1	I can never spend enough time on my mobile phone.	0.98	↑*	0.81	–	1.27	↑***	0.35	–
Item 2	I have used my mobile phone to make myself feel better when I was feeling down.	0.41	–	1.49	↑***	1.97	↑***	0.36	–
Item 3	I find myself occupied on my mobile phone when I should be doing other things, and it causes problems.	2.62	↑***	2.32	↑***	1.89	↑***	0.72	↑***
Item 4	All my friends own a mobile phone.	0.17	–	1.11	↑***	2.41	↑***	1.31	↑***
Item 5	I have tried to hide from others how much time I spend on my mobile phone.	1.82	↑***	1.93	↑***	1.28	↑***	0.37	↑*
Item 6	I lose sleep due to the time I spend on my mobile phone.	2.77	↑***	2.62	↑***	1.65	↑***	0.34	–
Item 7	I have received mobile phone bills I could not afford to pay.	−0.81	–	−0.14	–	0.41	–	−0.02	–
Item 8	When out of range for some time, I become preoccupied with the thought of missing a call.	0.22	–	0.19	–	0.18	–	0.29	–
Item 9	Sometimes, when I am on the mobile phone and I am doing other things, I get carried away with the conversation and I don't pay attention to what I am doing.	0.09	–	0.01	–	0.36	–	0.30	↑*
Item 10	The time I spend on the mobile phone has increased over the last 12 months.	−1.15	–	−0.10	–	0.01	–	0.19	–
Item 11	I have used my mobile phone to talk to others when I was feeling isolated.	0.57	–	1.41	↑**	1.99	↑***	0.71	↑*
Item 12	I have attempted to spend less time on my mobile phone but am unable to.	1.38	↑**	1.44	↑***	1.11	↑***	0.50	↑*
Item 13	I find it difficult to switch off my mobile phone.	0.16	–	0.99	↑*	0.79	↑*	0.36	–
Item 14	I feel anxious if I have not checked for messages or switched on my mobile	0.60	–	0.70	–	0.39	–	−0.02	–
Item 15	I have frequent dreams about the mobile phone.	0.69	↑**	0.64	↑*	0.24	–	0.09	–
Item 16	My friends and family complain about my use of the mobile phone.	0.58	–	1.32	↑***	0.88	↑*	0.31	–
Item 17	If I don't have a mobile phone, my friends would find it hard to get in touch with me	0.15	–	1.65	↑***	2.06	↑**	1.80	↑**
Item 18	My productivity has decreased as a direct result of the time I spend on the mobile phone.	2.54	↑***	2.05	↑***	1.34	↑***	0.48	↑*
Item 19	I have aches and pains that are associated with my mobile phone use.	0.67	↑*	1.29	↑***	0.79	↑**	0.28	–
Item 20	I find myself engaged on the mobile phone for longer periods of time than intended.	1.49	↑**	2.04	↑***	1.82	↑***	0.50	↑*
Item 21	There are times when I would rather use the mobile phone than deal with other more pressing issues.	2.85	↑***	2.88	↑***	2.30	↑***	0.62	↑**
Item 22	I am often late for appointments because I'm engaged on the mobile phone when I shouldn't be.	0.59	↑*	0.75	↑**	0.47	–	0.25	↑*
Item 23	I become irritable if I have to switch off my mobile phone for meetings, dinner engagements, or at the movies.	0.74	–	0.43	–	0.58	↑*	−0.15	–
Item 24	I have been told that I spend too much time on my mobile phone.	0.65	↑*	1.15	↑***	1.08	↑**	0.37	↑**
Item 25	More than once I have been in trouble because my mobile phone has gone off during a meeting, lecture, or in a theater.	0.12	–	−0.11	–	−0.03	–	−0.08	–
Item 26	My friends don't like it when my mobile phone is switched off.	−0.38	–	−0.14	–	−0.14	–	−0.40	–
Item 27	I feel lost without my mobile phone.	0.24	–	0.89	↑*	1.20	↑**	0.44	–

Δ = difference between item scores (2005 to 2018);

* p < 0.05;

** p < 0.01;

*** *p < 0.001*.

To illustrate changes in the prevalence of larger responses over time, the percentage of participants who marked a value of 6 or higher in each item was calculated for sex and age, as can be seen in Table A2. With regards to age, the 2018 sample consistently showed increases in selecting a value of 6 or higher for a majority of the MPPUS items.

Among the 18–25 years and 26–35 years age groups, the item with the highest increase in prevalence was Item 21 (“There are times when I would rather use the mobile phone than deal with other more pressing issues”). The proportion of participants who reported a score of 6 or higher for Item 21 in the 18–25 years old age group was 10.5% in 2005, and 51.4% in 2018. Likewise, the proportion of participants in 26–35 years old age group that reported scores of 6 or higher was 7.0% in 2005, and 44.8% in 2018. Among the 36–45 years old and 46+ years old age groups, the item with the highest increase in prevalence was Item 17 (“If I don't have a mobile phone, my friends would find it hard to get in touch with me”). The proportion of participants who reported a score of 6 or higher for Item 17 in the 36–45 years old group was 20.9% in 2005, and 52.0% in 2018. Additionally, the proportion of participants who reported a score of 6 or higher for Item 17 in the 46+ years old age group was 12.2% in 2005, and 36.0% in 2018.

With regards to sex, higher percentages of prevalence were also found in the 2018 sample, with more participants in this study selecting a value of 6 or higher for most of the MPPUS items. In the male population, the item with the largest increase in prevalence was Item 18 (“My productivity has decreased as a direct result of the time I spend on the mobile phone”), with the proportion of participants reporting a score of 6 or higher being 0% in 2005, and 12.5% in 2018. Amongst the female population, Item 17 (“If I don't have a mobile phone, my friends would find it hard to get in touch with me”) had the largest increase in prevalence, with the proportion of participants reporting a score of 6 or higher being 28.8% in 2005, and 54.9% in 2018.

A *t*-test was conducted between the 2018 Australian 26-item MPPUS scores and the 2005 Australian scores. There was a significant difference in the scores for the 2018 26-item MPPUS (*M* = 65.32, *SD* = 40.73) and the 2005 MPPUS (*M* = 57.08, *SD* = 30.66), such that the 2018 scores were higher [*t*_(402.078)_ = −3.08, *p* = 0.002].

## Discussion and Conclusion

The current study is a recent replication of the Mobile Phone Problem Use Scale (MPPUS) in an Australian sample. The first objective of this study was to investigate the current misuse of mobile phone technology in Australia and its potential implications on the health of Australians. Specifically, this study examined the relationship between problematic mobile phone use (at risk and problem users), sex, age, and education levels. The road safety implications of problematic mobile phone use were determined through the examination of the relationship between the MPPUS scores and mobile phone use while driving. Using a mobile phone while driving has been established as one of the riskiest behaviors with serious potential consequences such as property damage, injury, and death (40, 41). Additionally, differences were investigated between the scores of the current Australian sample and the original Australian sample studied by Bianchi and Phillips (29). This is the first replication study undertaken within Australia since Bianchi and Phillips' original research and confirmed the validity of the MPPUS to study problem mobile phone use. The findings of this paper offer new insights into the penetration of the role of mobile phones within a developed nation that has high mobile phone ownership rates.

The MPPUS scores were analyzed according to sex, showing that females report higher problematic phone use scores compared to males. Such findings are in line with previous research that has found greater mobile phone use among females, particularly in regards to females spending both a greater amount of time on their phone and money on their phone bill (42, 43). These results are also similar to a study conducted by Olivencia-Carrion et al. (44), whereby females were found to score higher in a questionnaire assessing mobile phone dependence based on addictive symptoms, time spent on the phone, etc. In addition, van Deursen et al. (45) also found that women, in comparison to men, were more likely to use their phones for social purposes. Both Nayak (42) and van Deursen et al. (45) attributed such findings to men experiencing less social stress than women. Furthermore, it is important to consider that these findings may be explained by differences in self-awareness, such that females tend to be more self-aware and self-report mobile phone use more openly (46). Contrary to expectations, the participants' sex did not predict Problematic Use which included those categorized as “At Risk” and “Problem Users,” compared with Normal Users which include “Casual Users” and “Habitual and Regular Users.” It is important to notice that these categories reflect common assumptions about the distribution of addictions in a population (30, 47). This finding mirrors that of Bianchi and Phillips (29) who did not find sex to be a predictor of smartphone usage, they found differences in the type of use, with females predominantly using their phones for social reasons and males for business purposes. More recent research has also found that females do not experience more problems related to mobile phone use compared to males (48, 49).

The results from this study also found significant differences between the three age groups, with the younger age group (18–24 years) reporting the highest levels of problematic mobile phone use, and the older age group (60+ years) reporting the lowest levels. This finding is in line with Bianchi and Phillips' (29) study, which found younger people to spend more time on their mobile phone and experience more problematic mobile phone use in comparison to older people. Similarly, these results are also consistent with another study which found younger participants (16–25 year-olds and 26–35 year-olds) to use their phones more problematically than the older participants (36 years and over), with hours of cell phone use diminishing with age (30). Overall, such findings highlight the possibility of younger people being more likely to embrace new technologies and utilize the various functions offered by mobile phones, such as SMS, social media, and gaming (29). Additionally, physical limitations such as issues with vision or manual dexterity may result in some older people using their phone less frequently in comparison to younger people (29). Generally, international findings have shown young people tend to be at higher risk of problematic phone use (50). An interesting finding was that there were significant associations between problematic mobile phone use and level of education, with participants enrolled or having completed university education reported higher MPPUS scores. This could be related to lifestyle variables not considered within this study.

This study found that there is a direct relationship between problematic mobile phone use and mobile phone use while driving. Specifically, self-reported handheld and hands-free mobile phone use increase with self-reported problematic mobile phone use. Participants who reported more handheld mobile phone use whilst driving are more likely to be classified as “problematic mobile phone users.” Research has been skeptical about defining mobile phone use as an addiction due to the lack of evidence for major consequences and symptomology of mobile phone use (51). As explained by Panova and Carbonell (6), one of the characteristics of addiction is the impairment of physical health, and in the case of mobile phone usage, physical consequences appear to be very minimal. The link between MPPUS and mobile phone use while driving suggests that problematic mobile phone use can indeed have physical consequences in the form of potential road crash-related injuries among drivers (52–54) and pedestrians (23). Data from naturalistic studies have found that the likelihood of a crash increases nearly 3.6 times as a result of handheld interactions with a mobile phone while driving (41). Specifically, in Australia, mobile phone use while driving is an activity that has been the target of a plethora of interventions to prevent these behaviors such as legislation, active police enforcement, educational campaigns, etc. However, these efforts have been mostly unsuccessful due to the ubiquitous nature of mobile phone use, lifestyle factors, and strategies to avoid police enforcement (55, 56). The current investigation adds to these factors, confirming that individual differences in the levels of problematic mobile phone use are related to mobile phone use while driving.

The results revealed that there are differences between MPPUS scores in 2005 and 2018, thus indicating that problem mobile phone use has, in fact, increased in Australia over the past 13 years. However, it is important to notice that this increased problem mobile phone use was not only among the younger generations but also participants aged 46 years old or more. As reported in the Deloitte (2) study, older generations have driven the market growth in Australia to 88% smartphone ownership. Further evidence of this phenomenon was seen when analyzing differences between self-reports in 2005 and 2018 in item 4 “All my friends own a mobile phone,” in which males, females and those aged 26 years old and older reported increased penetration of mobile technology in their social circles. This finding potentially signals the growing adaptation and versatility of mobile phone technology to support lifestyles across generations.

Among the most significant changes in item scores, Australians in 2018 reported experiencing more problems when occupied on their mobile phones, in addition to feeling more lost when their mobile phones are not in their possession. These findings may potentially be likened to the symptoms of tolerance, withdrawal and loss of control commonly discussed in literature on addictions, with the caveat that these symptoms in the context of mobile phones can be attributed to the substantial material cost, and the high level of integration of mobile phones within daily communication and social functioning (6).

Another interesting pattern is that of “technoference,” which in this study corresponds to the everyday intrusions and interruptions due to mobile phone devices and their usage (57). In this 2018 sample, technoference is shown to have increased amongst males and females across all ages. For example, self-reports of items such as “I lose sleep due to the time I spend on my mobile phone” (Item 6) and “my productivity has decreased as a direct result of the time I spend on the mobile phone” (Item 18) significantly increased during the last 13 years. This finding suggests that mobile phones are potentially increasingly affecting aspects of daytime functioning due to lack of sleep and increasing dereliction of responsibilities. These results confirm previous research that has linked mobile phone use with sleep and productivity disturbances (19, 58). An emerging explanation for the maladaptive relationship that individuals have with their mobile phone is “fear of missing out” (FOMO). FOMO is a psycho-social construct that is defined as the persistent desire to stay connected with others' rewarding experiences, and has been linked to both negative affectivity (e.g., stress, depression, anxiety) and increased severity of problem mobile phone use (59–61). While such findings are promising, more research into the cognitive impacts of smartphone technology is required to reach more definitive conclusions (62).

The non-significant differences between 2005 and 2018 responses suggest that some degree of tolerance or better community understanding of mobile phone devices has been achieved in the last 13 years. Examples of this are items such as “I have received mobile phone bills I could not afford to pay (Item 7),” “Sometimes, when I am on the mobile phone and I am doing other things, I get carried away with the conversation and I don't pay attention to what I am doing (Item 9),” and “The time I spend on the mobile phone has increased over the last 12 months (Item 10),” which did not increase. For the behavioral addictions, there has been some discussion as to whether increased access/exposure will always lead to problems [e.g., (63)] or whether a community eventually adapts [e.g., (64)]. Therefore, caution should be exercised in pathologizing the overall increased mobile phone ownership and usage, since professional, social, and academic contexts could heavily influence the frequency and type of certain mobile phone behaviors (6, 65). Nonetheless, an increased frequency of general phone use (regardless of being problematic or not) could result in a number of health issues such as musculoskeletal disorders (66).

In 2018, compared with the 2005 sample, more users in the 18–25 and 26–35-years old age groups reported that there were times when they would rather use their mobile phone than deal with other more pressing issues. This may indicate that mobile phones are also being used as a coping strategy that may facilitate avoidance of other “pressing issues” in one's life. Taken as a whole, this notable finding suggests that in spite of an increase in mobile phone use, not all phone use should be considered “bad” or problematic by nature, given that some individuals could be using the device in a way to better function during difficult times. Research conducted by Gonzales (67) particularly showed that disadvantaged groups (ethnic and sexual minorities) use the internet to enhance their social networks, with mobile phones being a key medium to facilitate this. The potential positive benefits of mobile phones must also be considered and contextualized when considering the overall impact of mobile phone technology.

### Limitations

The current study possesses some limitations that must be acknowledged. First, given the use of self-report data in the study, it is plausible that some participants may have overestimated their own mobile phone usage (30, 68). This may have influenced the potential accuracy of the findings in the study. To address this limitation, future research may look into using more objective measures of problem mobile phone use (e.g., apps which monitor frequency of use) and, if possible, gathering additional data from the people closest to the participant (e.g., family, colleagues) to strengthen the findings and generate a more holistic and accurate understanding of an individual's problem mobile phone use (52). Second, the unequal age and gender distributions resulted in this study comprising of a relatively older group of participants and, to a lesser extent, more males than females. This may have limited the study's ability to generalize its findings within a wider population of Australian mobile phone users. Third, this study did not investigate the health implications associated with mobile phone use while driving. Such information would have been of value within the field of road safety and can be considered an avenue for future research. The researchers also acknowledge that, due to rapid advancements in technology over the past decade, the ways in which people used mobile phones in 2005 is starkly different to the ways in which it is used in 2018. Consequently, our capacity to understand the mechanisms for the increase in the MPPUS scores is limited. Finally, it is important to note that the results may have potentially been affected due to differences in sample distributions between both the 2005 and 2018 surveys. The 2018 sample is a larger and more diverse sample regarding age and gender, and more representative of the general Australian population.

### Future Research

Future research may look into recruiting an equal number of participants within the age and gender groups. Finally, although mobile phone use seems to have increased in Australia, it is still unclear whether the maximum level has been reached as of yet. Further technological advancements such as the development of smart watches, for instance, are providing individuals with new and more accessible ways of utilizing the functions on their mobile phones without physically handling a phone in the first place. Thus, future research may investigate whether problematic use of such devices tends to exhibit similar health and safety implications to that of problematic mobile phone use.

## Data Availability

The datasets generated for this study are available on request to the corresponding author.

## Ethics Statement

This study was carried out in accordance with the recommendations of The National Statement on Ethical Conduct in Human Research. This study was approved by the QUT Human Research Ethics Committee (Approval no: 1800000170). All subjects gave informed consent in accordance with the Declaration of Helsinki.

## Author Contributions

OOT conceived the manuscript idea. All authors (OOT, SN, JDAN, DD, JGP) participated in the research and manuscript preparation.

### Conflict of Interest Statement

The authors declare that the research was conducted in the absence of any commercial or financial relationships that could be construed as a potential conflict of interest.
