# Supplementary material for: Problematic Use of Mobile Phones in Australia…Is It Getting Worse?
Source: Front Psychiatry. 2019 Mar 12;10:105. doi: 10.3389/fpsyt.2019.00105 (PMC6422909; doi:10.3389/fpsyt.2019.00105)
Supplement: Supplementary file 1 [file Data_Sheet_1.docx]

Appendix

Table A1. Exploratory factorial analysis to retest the validity of the MPPUS used in Australia in 2018

|  | | Items | | Component | | | | | |
| --- | --- | --- | --- | --- | --- | --- | --- | --- | --- |
|  | |  | | 1 | | 2 | | 3 | |
| Item 1 | | I can never spend enough time on my mobile phone. | | 0.693 | |  | |  | |
| Item 2 | | I have used my mobile phone to make myself feel better when I was feeling down. | | 0.715 | |  | |  | |
| Item 3 | | I find myself occupied on my mobile phone when I should be doing other things, and it causes problems. | | 0.809 | |  | |  | |
| Item 5 | | I have tried to hide from others how much time I spend on my mobile phone. | | 0.767 | |  | |  | |
| Item 6 | | I lose sleep due to the time I spend on my mobile phone. | | 0.781 | |  | |  | |
| Item 7 | | I have received mobile phone bills I could not afford to pay. | | 0.508 | |  | |  | |
| Item 8 | | When out of range for some time, I become preoccupied with the thought of missing a call | | 0.735 | |  | |  | |
| Item 9 | | Sometimes, when I am on the mobile phone and I am doing other things, I get carried away with the conversation and I don’t pay attention to what I am doing. | | 0.759 | |  | |  | |
| Item 10 | | The time I spend on the mobile phone has increased over the last 12 months. | | 0.601 | |  | |  | |
| Item 11 | | I have used my mobile phone to talk to others when I was feeling isolated. | | 0.639 | |  | |  | |
| Item 12 | | I have attempted to spend less time on my mobile phone but am unable to. | | 0.787 | |  | |  | |
| Item 13 | | I find it difficult to switch off my mobile phone. | | 0.762 | |  | |  | |
| Item 14 | | I feel anxious if I have not checked for messages or switched on my mobile phone for some time | | 0.763 | |  | |  | |
| Item 15 | | I have frequent dreams about the mobile phone. | | 0.614 | | 0.599 | |  | |
| Item 16 | | My friends and family complain about my use of the mobile phone. | | 0.728 | |  | |  | |
| Item 17 | | If I don’t have a mobile phone, my friends would find it hard to get in touch with me | | 0.433 | | -0.433 | | 0.428 | |
| Item 18 | | My productivity has decreased as a direct result of the time I spend on the mobile phone. | | 0.793 | |  | |  | |
| Item 19 | | I have aches and pains that are associated with my mobile phone use. | | 0.704 | |  | |  | |
| Item 20 | | I find myself engaged on the mobile phone for longer periods of time than intended. | | 0.785 | |  | |  | |
| Item 21 | | There are times when I would rather use the mobile phone than deal with other more pressing issues. | | 0.791 | |  | |  | |
| Item 22 | | I am often late for appointments because I’m engaged on the mobile phone when I shouldn’t be. | | 0.732 | |  | |  | |
| Item 23 | | I become irritable if I have to switch off my mobile phone for meetings, dinner engagements, or at the movies. | | 0.708 | |  | |  | |
| Item 24 | | I have been told that I spend too much time on my mobile phone. | | 0.786 | |  | |  | |
| Item 25 | | More than once I have been in trouble because my mobile phone has gone off during a meeting, lecture, or in a theatre. | | 0.626 | | 0.495 | |  | |
| Item 26 | | My friends don’t like it when my mobile phone is switched off. | | 0.529 | |  | | 0.42 | |
| Item 27 | | I feel lost without my mobile phone. | | 0.642 | |  | | 0.449 | |

*Note.* The principal component extraction method was used. Item 4 was removed for lack of signifincace.

Table A2. Percentage of Participants Who Marked a Value of Six or Higher in Each MPPUS Item (Based on Age and Sex) between 2005 and 2018

| Items | Age Groups | | | | | | | | Sex | | | | |
| --- | --- | --- | --- | --- | --- | --- | --- | --- | --- | --- | --- | --- | --- |
|  | 18-25 years | | 26-35 years | | 36-45 years | | 46+ years | | Male | | Females | | |
|  | 2005 | 2018 | 2005 | 2018 | 2005 | 2018 | 2005 | 2018 | 2005 | 2018 | 2005 | 2018 |  |
| Item 1 | 5.3% | 13.8% | 1.4% | 13.8% | 0.0% | 12.2% | 0.0% | 1.6% | 1.6% | 5.8% | 1.5% | 8.1% |  |
| Item 2 | 42.1% | 44.0% | 22.5% | 36.2% | 7.0% | 21.4% | 4.9% | 6.0% | 9.7% | 12.9% | 24.2% | 25.3% |  |
| Item 3 | 13.2% | 45.9% | 7.0% | 33.6% | 2.3% | 15.3% | 0.0% | 4.4% | 4.8% | 12.1% | 6.1% | 22.4% |  |
| Item 4 | 92.1% | 95.4% | 81.7% | 88.8% | 72.1% | 94.9% | 68.3% | 86.8% | 79.0% | 88.5% | 78.0% | 90.7% |  |
| Item 5 | 10.5% | 22.0% | 2.8% | 22.4% | 0.0% | 13.3% | 0.0% | 3.9% | 3.2% | 8.2% | 3.0% | 14.0% |  |
| Item 6 | 7.9% | 45.0% | 2.8% | 26.7% | 0.0% | 19.4% | 0.0% | 2.8% | 3.2% | 11.8% | 2.3% | 19.5% |  |
| Item 7 | 18.4% | 11.0% | 8.5% | 6.9% | 0.0% | 5.1% | 2.4% | 2.1% | 4.8% | 5.2% | 9.1% | 4.1% |  |
| Item 8 | 13.2% | 14.7% | 11.3% | 15.5% | 2.3% | 6.1% | 0.0% | 2.6% | 4.8% | 5.8% | 8.3% | 8.4% |  |
| Item 9 | 23.7% | 22.9% | 22.5% | 21.6% | 9.3% | 10.2% | 4.9% | 4.4% | 16.1% | 9.3% | 15.9% | 12.5% |  |
| Item 10 | 47.4% | 34.9% | 33.8% | 31.0% | 20.9% | 21.4% | 9.8% | 13.7% | 35.5% | 17.8% | 25.0% | 24.1% |  |
| Item 11 | 42.1% | 56.0% | 38.0% | 52.6% | 4.7% | 31.6% | 2.4% | 9.8% | 12.9% | 17.8% | 29.5% | 36.6% |  |
| Item 12 | 10.5% | 22.0% | 5.6% | 28.4% | 2.3% | 14.3% | 0.0% | 4.1% | 4.8% | 10.1% | 4.5% | 14.5% |  |
| Item 13 | 34.2% | 31.2% | 25.4% | 43.1% | 16.3% | 25.5% | 4.9% | 9.3% | 17.7% | 16.7% | 22.0% | 24.4% |  |
| Item 14 | 21.1% | 33.9% | 22.5% | 36.2% | 14.0% | 16.3% | 7.3% | 7.8% | 16.1% | 14.0% | 17.4% | 21.5% |  |
| Item 15 | 2.6% | 9.2% | 0.0% | 6.0% | 0.0% | 0.0% | 0.0% | 0.5% | 1.6% | 3.3% | 0.0% | 2.0% |  |
| Item 16 | 13.2% | 17.4% | 0.0% | 15.5% | 0.0% | 9.2% | 0.0% | 3.1% | 1.6% | 7.9% | 3.0% | 8.4% |  |
| Item 17 | 60.5% | 66.1% | 38.0% | 68.1% | 20.9% | 52.0% | 12.2% | 36.0% | 41.9% | 41.6% | 28.8% | 54.9% |  |
| Item 18 | 5.3% | 33.0% | 1.4% | 26.7% | 0.0% | 14.3% | 0.0% | 3.4% | 0.0% | 12.6% | 2.3% | 14.0% |  |
| Item 19 | 7.9% | 11.9% | 4.2% | 17.2% | 0.0% | 9.2% | 0.0% | 2.8% | 3.2% | 6.6% | 3.0% | 8.4% |  |
| Item 20 | 23.7% | 46.8% | 21.1% | 44.0% | 7.0% | 27.6% | 2.4% | 7.5% | 17.7% | 16.4% | 12.9% | 28.5% |  |
| Item 21 | 10.5% | 51.4% | 7.0% | 44.8% | 0.0% | 21.4% | 0.0% | 4.7% | 6.5% | 15.9% | 3.8% | 25.9% |  |
| Item 22 | 7.9% | 11.9% | 4.2% | 9.5% | 0.0% | 3.1% | 0.0% | 1.3% | 3.2% | 4.7% | 3.0% | 4.4% |  |
| Item 23 | 2.6% | 12.8% | 7.0% | 7.8% | 0.0% | 5.1% | 4.9% | 1.6% | 6.5% | 4.7% | 3.0% | 4.9% |  |
| Item 24 | 18.4% | 22.0% | 4.2% | 16.4% | 2.3% | 15.3% | 0.0% | 2.1% | 3.2% | 8.5% | 6.8% | 10.2% |  |
| Item 25 | 13.2% | 11.9% | 12.7% | 11.2% | 7.0% | 6.1% | 2.4% | 2.1% | 14.5% | 5.5% | 6.8% | 5.8% |  |
| Item 26 | 31.6% | 25.7% | 25.4% | 25.9% | 4.7% | 9.2% | 9.8% | 6.7% | 16.1% | 12.9% | 19.7% | 13.4% |  |
| Item 27 | 28.9% | 33.9% | 21.1% | 35.3% | 16.3% | 27.6% | 4.9% | 12.7% | 11.3% | 17.8% | 21.2% | 25.9% |  |
